# Draft genome sequence of *Enterococcus faecalis* BFS29 isolated from diseased silver carp (*Hypophthalmichthys molitrix*) in Bangladesh

**DOI:** 10.1128/mra.01286-25

**Published:** 2026-03-27

**Authors:** Md. Rony Babu, Promi Saha, Md. Mahbub Alam, Sulav Indra Paul, Tasmina Akter, Alfred Chin Yen Tay, Md. Javed Foysal, Tofazzal Islam, Md. Mahbubur Rahman

**Affiliations:** 1 Institute of Biotechnology and Genetic Engineering, Gazipur Agricultural University 198780, Gazipur, Bangladesh; 2 Department of Fisheries, Government of the People’s Republic of Bangladesh, Dhaka, Bangladesh; 3 Institute for Biosecurity and Microbial Forensics, Oklahoma State University 7618, Stillwater, Oklahoma, USA; 4 Department of Fisheries Management, Gazipur Agricultural University 198780, Gazipur, Bangladesh; 5 Marshall Centre for Infectious Diseases Research and Training, Helicobacter Research Laboratory, University of Western Australia 2720, Perth, Australia; 6 Department of Genetic Engineering and Biotechnology, Shahjalal University of Science and Technology 113074, Sylhet, Bangladesh; 7 Faculty of Medicine and Health, The University of Sydney 4334, Sydney, Australia; Portland State University, Portland, Oregon, USA

**Keywords:** *Enterococcus faecalis*, silver carp, genome sequence, virulence genes, antimicrobial resistance

## Abstract

We report a draft genome sequence of *Enterococcus faecalis* BFS29, isolated from a diseased silver carp in Bangladesh. The 2.89 Mb genome assembled into 69 contigs with a GC content of 37.4%, encodes 2,789 predicted coding sequences, and contains virulence, antimicrobial resistance, and bacteriocin-associated genes.

## ANNOUNCEMENT

*Enterococcus faecalis* is a species of gram-positive, facultatively anaerobic bacterium commonly inhabiting the gastrointestinal tracts of humans and animals (1). It can also infect immunocompromised hosts (2). In aquaculture, it causes septicemia, exophthalmia, and mortality in tilapia (3, 4), catfish (3), silver barb (5), and mullet (6), leading to significant economic losses.

Strain BFS29 was isolated from the liver of a silver carp (*Hypophthalmichthys molitrix*) suffering from a streptococcosis-like infection in a freshwater pond in Zakiganj, Sylhet, Bangladesh, in July 2011. The fish exhibited bilateral eye opaqueness, excessive mucus secretion, fin and skin erosion, and erratic swimming behavior. A homogenate of the liver was serially diluted, plated on Kenner-Faecal streptococcal agar plates (HiMedia, India), and incubated at 35°C for 24 h (5). BFS29 was picked up after multiple rounds of single-colony isolation and preserved in 12 h nutrient broth culture with 10% glycerol at −80°C. The preserved sample was thawed and inoculated on nutrient agar, and a single colony was grown in nutrient broth at 28°C with shaking (120 rpm) for 24 h (3). Genomic DNA was extracted (from 5 × 10⁸ cfu/mL) using the GeneJET genomic DNA purification kit (Thermo Fisher Scientific, USA). DNA quality and concentration were assessed using a NanoDrop spectrophotometer (Thermo Scientific, USA) and a Qubit fluorometer (Thermo Scientific, USA). A sequencing library was prepared from 1 ng DNA using Illumina Nextera XT kit, following tagmentation at 55°C for 10 min and 12 cycles of barcoding PCR (7, 8). The library was purified with AMPure XP beads, normalized to 5 nM, denatured using 0.2 N NaOH, diluted to 13 pM, and sequenced on an Illumina MiSeq platform (600-cycle run).

Raw reads (3,513,660) with a coverage of 30× were processed using the Bacterial Analysis Pipeline v1.0.4 (9), trimmed with Trimmomatic v0.38 (10), and filtered with PRINSEQ v0.20.3 (11). *De novo* assembly was conducted with SPAdes v3.9.0 (12), and assembly quality was evaluated with QUAST v5.0.2 (13). Default parameters were used for bioinformatics tools unless otherwise specified. The assembly resulted in 69 contigs with an estimated chromosome size of 2,890,867 bp and 37.4% GC content. Assembly metrics included an N50 of 141,195 bp, L50 of 7, and the largest contig of 399,482 bp. Annotation using the NCBI Prokaryotic Genome Annotation Pipeline (PGAP, v6.10) (14) identified 2,859 genes, including 2,789 coding sequences (2,757 protein-coding), 70 RNAs (55 tRNAs, 4 ncRNAs, and 11 rRNAs), 32 pseudogenes, and 2 CRISPR arrays. RAST analysis (FIGfams v70) (15) revealed 244 functional subsystems, representing 29% of the genome. Virulence Factor Database (16) analysis identified nine virulence genes (*ace*, *ebpA*, *efaA*, *cpsA/uppS*, *bopD*, *fsrA*, *gelE*, and *sprE*), associated with adhesion, biofilm formation, enzymatic activity, and serum resistance. ResFinder (17) identified three acquired antimicrobial resistance genes: *lsa(A)* (lincosamide resistance), *tet(M*), and *tet(L)* (tetracycline resistance). BAGEL4 analysis (18) predicted two bacteriocin biosynthesis clusters, encoding propeptin-like and enterocin SE-K4-like peptides.

The draft genome of BFS29 provides valuable insights on virulence and antimicrobial resistance in fish disease-associated *E. faecalis*, supporting future studies on host adaptation, diagnosis, and control strategies in aquaculture.

## Data Availability

The draft genome sequence of *E. faecalis* BFS29 has been deposited in GenBank under accession number JBNOXS000000000.1. The associated BioProject and BioSample identifiers are PRJNA1258581 and SAMN48325304, respectively. The Sequence Read Archive (SRA) provides access to raw sequencing reads using the accession number SRX28659865.

## References

[1] Boeder AM, Spiller F, Carlstrom M, Izídio GS. 2024. Enterococcus faecalis: implications for host health. World J Microbiol Biotechnol 40:190. doi:10.1007/s11274-024-04007-w 38702495

[2] Ramos S, Silva V, Dapkevicius MDLE, Igrejas G, Poeta P. 2020. Enterococci, from harmless bacteria to a pathogen. Microorganisms 8:1118. doi:10.3390/microorganisms8081118 32722391 PMC7463792

[3] Rahman M, Rahman MM, Deb SC, Alam MS, Alam MJ, Islam MT. 2017. Molecular Identification of multiple antibiotic resistant fish pathogenic Enterococcus faecalis and their control by medicinal herbs. Sci Rep 7:3747. doi:10.1038/s41598-017-03673-1 28623336 PMC5473830

[4] Akter T, Foysal MJ, Alam M, Ehsan R, Paul SI, Momtaz F, Siddik MAB, Tay ACY, Fotedar R, Gupta SK, Islam T, Rahman MM. 2021. Involvement of Enterococcus species in streptococcosis of Nile tilapia in Bangladesh. Aquaculture 531:735790. doi:10.1016/j.aquaculture.2020.735790

[5] Ehsan R, Alam M, Akter T, Paul SI, Foysal MJ, Gupta DR, Islam T, Rahman MM. 2021. Enterococcus faecalis involved in streptococcosis like infection in silver barb (Barbonymus gonionotus). Aquaculture Reports 21:100868. doi:10.1016/j.aqrep.2021.100868

[6] Hassan MA, Abdel‐Naeim NS, Mabrok M, Dessouki AA, Hassan AM. 2022. Isolation and identification of Enterococcus faecalis from cultured Oreochromis niloticus and Mugil cephalus with a special emphasis on a possible integrated control strategy. Aquac Res 53:5521–5535. doi:10.1111/are.16034

[7] Akter T, Rahman MM, Tay ACY, Ehsan R, Islam MT. 2020. Whole-genome sequence of fish-pathogenic Enterococcus faecalis strain BFFF11. Microbiol Resour Announc 9:01447–19. doi:10.1128/MRA.01447-19 PMC7019064 32054709

[8] Illumina. 2014. Nextera® XT DNA library preparation guide. https://genome.med.harvard.edu/documents/libraryPrep/IlluminaNexteraXTProtocol.pdf.

[9] Larsen MV, Cosentino S, Lukjancenko O, Saputra D, Rasmussen S, Hasman H, Sicheritz-Pontén T, Aarestrup FM, Ussery DW, Lund O. 2014. Benchmarking of methods for genomic taxonomy. J Clin Microbiol 52:1529–1539. doi:10.1128/JCM.02981-13 24574292 PMC3993634

[10] Bolger AM, Lohse M, Usadel B. 2014. Trimmomatic: a flexible trimmer for Illumina sequence data. Bioinformatics 30:2114–2120. doi:10.1093/bioinformatics/btu170 24695404 PMC4103590

[11] Schmieder R, Edwards R. 2011. Quality control and preprocessing of metagenomic datasets. Bioinformatics 27:863–864. doi:10.1093/bioinformatics/btr026 21278185 PMC3051327

[12] Bankevich A, Nurk S, Antipov D, Gurevich AA, Dvorkin M, Kulikov AS, Lesin VM, Nikolenko SI, Pham S, Prjibelski AD, Pyshkin AV, Sirotkin AV, Vyahhi N, Tesler G, Alekseyev MA, Pevzner PA. 2012. SPAdes: a new genome assembly algorithm and its applications to single-cell sequencing. J Comput Biol 19:455–477. doi:10.1089/cmb.2012.0021 22506599 PMC3342519

[13] Gurevich A, Saveliev V, Vyahhi N, Tesler G. 2013. QUAST: quality assessment tool for genome assemblies. Bioinformatics 29:1072–1075. doi:10.1093/bioinformatics/btt086 23422339 PMC3624806

[14] Tatusova T, DiCuccio M, Badretdin A, Chetvernin V, Nawrocki EP, Zaslavsky L, Lomsadze A, Pruitt KD, Borodovsky M, Ostell J. 2016. NCBI prokaryotic genome annotation pipeline. Nucleic Acids Res 44:6614–6624. doi:10.1093/nar/gkw569 27342282 PMC5001611

[15] Overbeek R, Olson R, Pusch GD, Olsen GJ, Davis JJ, Disz T, Edwards RA, Gerdes S, Parrello B, Shukla M, Vonstein V, Wattam AR, Xia F, Stevens R. 2014. The SEED and the rapid annotation of microbial genomes using subsystems technology (RAST). Nucleic Acids Res 42:D206–14. doi:10.1093/nar/gkt1226 24293654 PMC3965101

[16] Liu B, Zheng D, Jin Q, Chen L, Yang J. 2019. VFDB 2019: a comparative pathogenomic platform with an interactive web interface. Nucleic Acids Res 47:D687–D692. doi:10.1093/nar/gky1080 30395255 PMC6324032

[17] Zankari E, Hasman H, Cosentino S, Vestergaard M, Rasmussen S, Lund O, Aarestrup FM, Larsen MV. 2012. Identification of acquired antimicrobial resistance genes. J Antimicrob Chemother 67:2640–2644. doi:10.1093/jac/dks261 22782487 PMC3468078

[18] van Heel AJ, de Jong A, Song C, Viel JH, Kok J, Kuipers OP. 2018. BAGEL4: a user-friendly web server to thoroughly mine RiPPs and bacteriocins. Nucleic Acids Res 46:W278–W281. doi:10.1093/nar/gky383 29788290 PMC6030817

